# Inversion of Spin Signal and Spin Filtering in Ferromagnet|Hexagonal Boron Nitride-Graphene van der Waals Heterostructures

**DOI:** 10.1038/srep21168

**Published:** 2016-02-17

**Authors:** M. Venkata Kamalakar, André Dankert, Paul J. Kelly, Saroj P. Dash

**Affiliations:** 1 Department of Microtechnology and Nanoscience, Chalmers University of Technology, SE-41296, Göteborg, Sweden; 2 Department of Physics and Astronomy, Uppsala University, Box 516, 75120, Uppsala, Sweden; 3 Faculty of Science and Technology and MESA^+^ Institute for Nanotechnology, University of Twente, P.O. Box 217, 7500 AE Enschede, The Netherlands

## Abstract

Two dimensional atomically thin crystals of graphene and its insulating isomorph hexagonal boron nitride (h-BN) are promising materials for spintronic applications. While graphene is an ideal medium for long distance spin transport, h-BN is an insulating tunnel barrier that has potential for efficient spin polarized tunneling from ferromagnets. Here, we demonstrate the spin filtering effect in cobalt|few layer h-BN|graphene junctions leading to a large negative spin polarization in graphene at room temperature. Through nonlocal pure spin transport and Hanle precession measurements performed on devices with different interface barrier conditions, we associate the negative spin polarization with high resistance few layer h-BN|ferromagnet contacts. Detailed bias and gate dependent measurements reinforce the robustness of the effect in our devices. These spintronic effects in two-dimensional van der Waals heterostructures hold promise for future spin based logic and memory applications.

Two dimensional (2D) crystals of graphene (Gr) and hexagonal boron nitride (h-BN) have gained immense attention in recent years because of their exciting properties for nanoelectronics^1^,^2^,^3^ and spintronics^4^,^5^,^6^,^7^,^8^,^9^,^10^. In particular, graphene is an ideal medium for long distance spin transport due to the low atomic mass of carbon atoms with zero net nuclear spin^4^,^11^. On the other hand, h-BN is an insulator with a bandgap of ~6 eV, devoid of interface states^12^, which makes it an ideal material for atomically thin tunnel barrier applications^2^,^9^,^13^. In addition to their stand-alone impact, it is possible to create van der Waals heterostructures (vdWHs)^14^ by combining these 2D crystals in stacks. The structural compatibility of graphene and h-BN (lattice mismatch of only 1.7%) together with their complementary electrical properties makes them excellent partners for vdWHs^14^. While such vdWHs serve as unique test beds for studying interesting physical phenomenon, they can also be utilized in devices to achieve enhanced performance^14^. For instance, long spin coherence lengths in graphene have been achieved by using h-BN as a substrate and for encapsulating graphene^15^,^16^,^17^,^18^.

Recently h-BN has been shown to be a promising 2D material for tunnel barrier applications^2^. Atomically thin h-BN has also been demonstrated as a promising spin tunnel barrier material for spin injection from a ferromagnet (FM) into graphene^5^,^9^,^19^,^20^ and for magnetic tunnel junctions^21^. Such tunnel contacts are essential for efficient spin injection as they help to overcome the conductivity mismatch problem between the metallic ferromagnet and graphene^22^. In general, the spin polarization or the difference in population of up (↑) and down (↓) spin density from a ferromagnetic injector can decay inside an ultrathin tunnel barrier to some extent. However, a ferromagnetic junction with novel crystalline barriers can present spin resolved conductance that can further influence the magnitude and sign of the spin polarization from ferromagnets^6^,^23^. The 2D crystals are a new class of barrier materials, significantly different from conventional oxide barriers in many aspects, such as their atomically thin nature, absence of dangling bonds and van der Waals forces that play key role in their intra layer bonding and interface bonding with other 2D crystals and materials. Theoretically, 2D crystals of h-BN and graphene in contact with ferromagnets have been predicted to show spin resolved conductivities 6,7,8,10,23,24. These reports indicate that 2D h-BN is a novel spin tunnel barrier with the potential for spin filtering in FM|h-BN|Gr junctions leading to high negative spin polarization. However, owing to the challenges involved in device fabrication and required contact interface conditions, such spin filtering in FM|h-BN|Gr interfaces is yet to be observed experimentally.

In this article we report an inversion of spin signal with large magnitude of spin polarization in non-local graphene spin valves with h-BN|cobalt (Co) high resistance (HR)–low resistance (LR) injector-detector (with different h-BN thickness) combination tunnel contacts. Through systematic nonlocal pure spin transport and Hanle spin precession measurements on devices having different interface barrier conditions, we associate the negative spin polarization with HR h-BN|Co contacts. The characteristic polarization and spatial spin filtering across such contacts leads to relatively large magnitude of spin signal in these devices. We further establish the robustness of the sign of polarization through bias and gate dependent measurements. Our results show the possibility of manipulating the sign and magnitude of spin polarization due to different interface conditions with thickness of h-BN barriers. The large and negative spin polarization using such high resistance (HR) h-BN|Co contacts observed in graphene non-local spin valves indicates spin filtering effects and opens avenues for efficient 2D vdWH spintronic devices.

## Results

**Figure 1:**
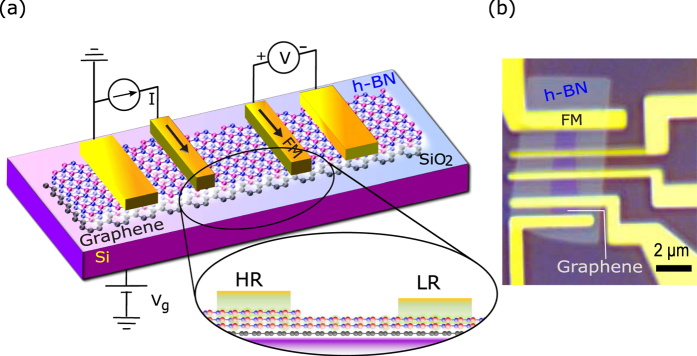
Ferromagnet|Hexagonal Boron Nitride-Graphene van der Waals heterostructure spin transport devices. (**a**) Schematic representation of the h-BN|graphene van der Waals heterostructures (vdWH) with multiple ferromagnetic (FM) contacts for spin injection and detection in nonlocal geometry. The magnified view of the contacts exemplifies different thicknesses of h-BN barriers under the high resistance (HR) injector and low resistance (LR) detector. (**b**) Optical microscope image (color enhanced) of a fabricated multi-terminal spin transport device showing a heterostructure of a graphene flake covered by a CVD h-BN tunnel barrier and multiple ferromagnetic Co electrodes.

For spin transport measurements, we fabricated lateral graphene(Gr)|h-BN vdWH devices with ferromagnetic Co contacts. A schematic representation of realizing injection and detection contacts with different h-BN thicknesses along with an optical microscope image of an actual device is presented in Fig. 1. Spin accumulation in graphene is achieved by electrical spin injection from a Co|h-BN tunnel contact and the induced spin splitting (Δμ) is measured via nonlocal (NL) spin transport and precession methods (Fig. 1a). We used graphene flakes exfoliated from highly oriented pyrolitic graphite (HOPG) on Si|SiO_2_ substrates. A layer of CVD h-BN (Fig. S1) was transferred onto the graphene and subsequently annealed in Ar/H_2_ to achieve clean interfaces and improve adhesion in the vdWH. The ferromagnetic Co electrodes were prepared by electron-beam lithography and evaporation techniques (Fig. 1b). Details of the fabrication of the vdWH spintronic devices are presented in methods.

**Figure 2:**
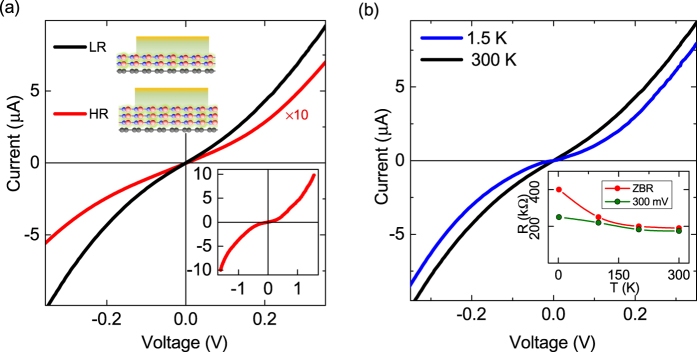
Electrical characterization of CVD h-BN tunnel barrier. **(a**) Three-terminal I-V characteristics of high resistance (HR) and low resistance (LR) h-BN (Co|h-BN|Graphene) contacts at room temperature. The current for the HR contact is scaled by a factor of 10 to highlight the non-linearity with bias. Inset: As measured I-V curve of the HR contact in a higher current-voltage range. (**b)** Temperature dependent I-V characteristics for the LR h-BN contact at 1.5 and 300 K. Inset: Temperature dependence of the HR h-BN contact resistance at zero bias (ZBR) and 300 mV.

For efficient spin injection into graphene, the tunneling nature of the h-BN barrier with appropriate contact resistance constitutes the prime requirement. To assess the quality of the Co|h-BN|graphene contacts, we performed temperature dependent current-voltage (I-V) characterization of the injection and detection contacts in a three-terminal configuration^9^. Figure 2a shows the non-linear I-V characteristics of the spin injection and detections contacts showing different resistance area products (RA): a high resistance (HR) injector (*R*_*i*_ = 170 kΩ μm^2^) and a low resistance (LR) detector (*R*_*d*_ = 25 kΩ μm^2^). Such a difference in contact resistance is a characteristic feature of CVD h-BN tunnel barriers due to a spatial thickness distribution, which is mostly 1–2 layers with some patches of higher thickness (~3 layers) as reported previously^9^,^19^. Even though very high resistance contacts with resistance ~0.5–1 MΩ are also found, our previous studies^9^ indicate that such contacts are unsuitable for spin injection/detection. Atomic force microscopy performed on post fabricated devices show the effective thickness of the h-BN layer to be mostly between 5–10 Å on SiO_2_ substrates (Supplementary Information Fig. S2). This thickness variation in CVD h-BN provides an opportunity to investigate spin injection using different thicknesses and hence different resistances of h-BN tunnel barriers. By optimizing the CVD h-BN transfer technique and subsequent processing, we ensured clean interfaces (see fabrication details in Supplementary Information), which is also reflected in our electric and spin transport characteristics. The temperature dependence of both HR and LR h-BN tunnel contacts was found to be weak in the range of 1.5–300 K (inset of Fig. 2b). The change in tunnel barrier resistance by less than a factor of 2 in the measured temperature range indicates a good quality of the insulating interface^25^, and ensures the viability of CVD h-BN as a reliable tunnel barrier.

**Figure 3:**
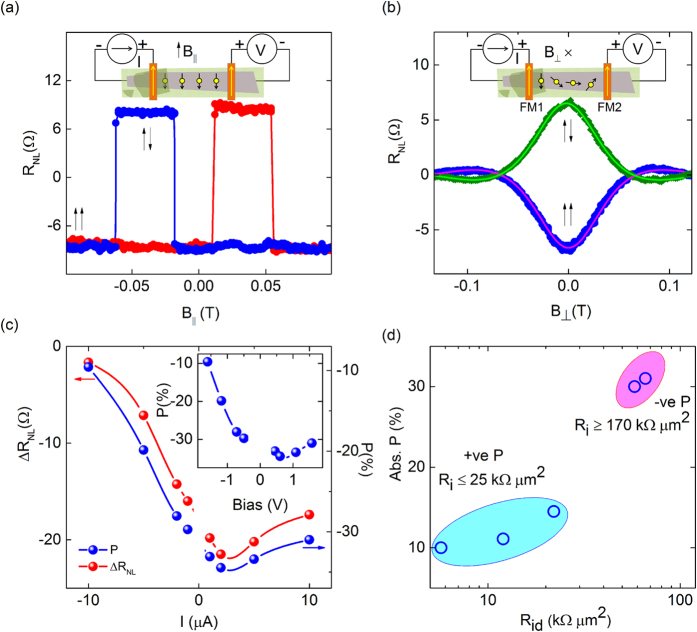
Spin transport and precession measurements at room temperature with spin filtering in Co|h-BN|Gr contact. (**a**) Nonlocal spin valve signal (*V_NL_*) measured at 300 K using an injection current of *I* = 10 μA with in-plane magnetic field. Magnetic field sweep directions are indicated by red (up) and blue (down) colors. (**b)** Nonlocal Hanle spin-precession signal measured as a function of the perpendicular magnetic field with *I* = 10 μA at 300 K, keeping the magnetization of the ferromagnetic electrodes in an in-plane parallel (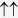) and anti-parallel (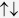) configuration. The solid line is the Hanle fit (Eq. 1) to the data points using Eq. 1 for a field T. The nonlocal resistance is defined as 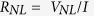
and 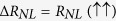 −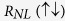. Insets in a and b show the spin valve and Hanle measurement configurations on graphene for a device with spin injection contact of a thicker layer of h-BN and detection under a thinner h-BN with Co electrodes. (**c)** Dependence of 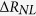
and spin polarization 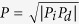 on the injection bias current at 300 K, P_i_ and P_d_ are injector and detector polarizations respectively. Inset: Dependence of P on the injection bias voltage at 300 K. The circles are the data points and the lines are guides to the eye. (**d)** Absolute value of the spin polarization 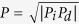 versus the tunnel contact resistance 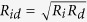 for different devices, *R*_*i*_ and *R*_*d*_ are injector and detector resistances. The sign of the spin polarization for the *R*_*id*_ involving high resistance h-BN contact is negative, whereas it is positive for lower resistance devices. *R*_*id*_ = 65 kΩ μm^2^ corresponds to devices with *R*_*i*_ = 170 kΩ μm^2^ and *R*_*d*_ = 25 kΩ μm^2^, *R*_*id*_ ≈ 25 kΩ μm^2^corresponds to a device with *R*_*i*_ ≈ *R*_*d*_ ≈ 25 kΩ. The spin polarization becomes negative when *R*_*i*_ increased from 25–170 kΩ μm^2^due to an effective increase in h-BN thickness.

To probe the spin transport using such Co|h-BN contacts on graphene, measurements were carried out in the four terminal nonlocal (NL) configuration as depicted in the inset of Fig. 3a. The spins are electrically injected between contacts +I and −I, and a NL voltage due to the spin accumulation is measured by the voltage circuit. The isolated current and voltage circuits in the NL configuration ensure the measurement of pure spin signals without spurious magneto-resistive contributions^4^. In the spin valve measurement configuration shown in the inset of Fig. 3a, we sweep an in-plane magnetic field to detect the spin signal (*V_NL_*) for parallel and anti-parallel configurations of the injection and detection ferromagnetic electrodes. This gives a measure of the induced spin splitting (Δμ) in graphene and hence the spin polarization of the Co|h-BN tunnel contacts. The different shape anisotropies of the ferromagnetic contacts ensure distinct switching fields for each of them. In contrast to the normally observed NL spin signal in graphene spin valves (shown in Supplementary Information Fig. S3), we observed an inverted signal in our devices that have a high resistance (HR) injection or detection contact. A normal sign of the spin signal corresponds to similar positive nature of the spin polarization of both junctions. On the other hand, the inverted signal points to an opposite sign of the spin polarizations of the injector (+I) and detector (+V) contacts. A possible negative spin polarization in one of the Co|h-BN|Gr junctions can yield an inverted spin signal. Variation in barrier thickness and corresponding resistance in Co|h-BN|Gr contacts can give rise to different spin polarizations^23^. Notably, the sign and magnitude of the spin signal remained similar when the injector and detector were interchanged during measurements. Similar inverted spin-valve signals have been reproducibly observed in devices specifically involving HR h-BN contacts.

To confirm the nature and magnitude of the spin polarization of the Co|h-BN tunnel contacts and to estimate the spin lifetime in graphene, we performed Hanle spin precession measurements in the NL configuration^4^. In the Hanle geometry, a spin signal is modulated (Δ*R*_*NL*_) by spin precession in a perpendicular magnetic field (Fig. 3b). In contrast to the regular Hanle signal obtained for a parallel configuration of injector and detector (shown in Supplementary Information Fig. S3), we obtained an inverted Hanle spin signal. This further supports our observation of inverted spin signal in spin valve measurements and confirms that the observed effect is not related to any unusual switching of the ferromagnetic tunnel contacts. The inverted spin signal in both the NL spin valve and Hanle measurements provides evidence that the Co|h-BN|Gr tunnel contacts can produce negative spin polarization. The variation in Hanle *R*_*NL*_ is well described by the expression Eq. (1) which encompasses spin diffusion, precession and dephasing contributions.


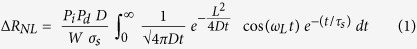


where *P*_*i*_ and *P*_*d*_ are the polarizations for an injector and a detector separated by a channel with effective length *L* = *1.93 μm,* width *W* = *1.05 μm* and graphene square conductivity *σ*_*S*_ = 340 *μS.* The spins undergo Larmor precession with frequency 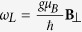 (with Lande g-factor g = 2) in the perpendicular magnetic field 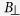 while diffusing across the channel. From the fitting of our experimental data (Fig. 3b), we obtain a spin lifetime 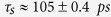, and a diffusion constant *D* ≈ 0.005 ± 0.0003 m^2^s^−1^ matching closely with the charge diffusion constant (0.006 m^2^s^−1^). We have also found similar spin lifetime in graphene devices prepared with TiO_2_ tunnel barrier and in other studies^4^,^26^,^27^ on graphene with moderate mobilities ~1000 cm^2^V^−1^s^−1^. The actual tunnel resistances (not Resistance × contact Area) in our device *R*i** and *R_d_* >> 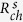 graphene spin resistance ~5 kΩ, (where *R_i_* and *R_d_* are the actual contact resistances of injector and detector contacts and the channel spin resistance^28^,^29^ 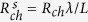 for channel with length *L* and resistance 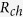), large enough to rule out any contact induced relaxation and spin sink effects^30^,^31^ (see Fig. S4). This indicates that the 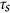 in our devices is mainly affected by the spin relaxation in the graphene channel due to impurities and defects.

From our analysis, using Eq. 1 we determine a net tunnel spin polarization 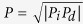  ≈ 31% for the Co|h-BN contacts at room temperature. Such a value of *P* is considered to be quite large for graphene spin valve devices^32^,^33^. Figure 3c and inset show the variation of *ΔR_NL_*and *P* with injection bias current and voltages, exhibiting a decreasing trend at higher bias. It is worth mentioning that the spin lifetime and diffusion constant were seen to be fairly independent of the bias (Supplementary Information Fig. S4a). Since these parameters are physically independent of polarization *P*, their values do not influence the estimation of *P*. Also, to note, as the detector was not biased in our experiments, *P*_*d*_ remains fairly constant and only the HR contact *P*_*i*_ can determine this behavior. The observed decreasing trend of *P* for higher bias voltages is a characteristic feature of magnetic tunnel junctions. Such behavior in *P* can be ascribed to energy dependence of the spin polarization, electric field effects, magnon excitations^34^, and spin exchange scattering due to defects^35^ at the interface. An asymmetric bias dependence in spin polarization (as shown in Fig. 3c) can also be expected for asymmetric junctions (in our case Co|h-BN|Gr) for spin polarized tunneling at finite bias^36^. The asymmetry can be attributed to the intrinsic barrier height asymmetry and available/occupied density of states for spin transmission from Co to graphene and vice-versa^36^, with different spin selectivity and polarization^35^,^36^. Such non-linear bias dependence of polarization has been previously observed in graphene based devices^33^ and in magnetic tunnel junctions^34^,^37^,^38^. We did not observe any appreciable change in the polarization with temperatures down to 100 K.

To investigate the dependence of *P* on the h-BN contact resistances, a number of graphene spin valve devices were measured. A systematic variation of *P* with effective h-BN barrier resistance (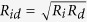) is presented in Fig. 3d. We observed an enhancement in the magnitude of *P* with effective barrier resistance 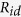 of the devices. The highest spin polarization of 31% (negative sign) belongs to the high resistance h-BN device having *R*_*id*_ = 65 kΩ μm^2^ with *R*_*i*_ = 170 kΩ μm^2^ and 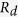 = 25 kΩ μm^2^. Practically, the thicker h-BN barrier can also provide better interfaces and alleviate the problems of possible diffusion of ferromagnetic impurities and hence can lead to better polarization. In this study, we chose to compare devices prepared on the same Si/SiO_2_ substrate, under the same conditions, showing similar mobility and spin parameters to understand the variation of polarization with barrier resistance. Since the spin polarization and lifetime are independent parameters, using high quality graphene channel with better spin parameters^11^,^17^,^18^ can lead to enhancements in *ΔR_NL_* much beyond 20 Ω. We note that the observed large spin polarization and its negative nature are found to occur only when the contact resistance changes by a large magnitude (*R*_*i*_ from 25 to 170 kΩ μm^2^) due to an effective increase in the thickness of h-BN by one layer^2^,^9^,^19^. Noting that the spin polarization of the LR Co/h-BN detector contact *P*_*d*_ ≈ 15% (with *R*_*id*_ ≈ 25 kΩ μm^2^ from Fig. 3d), with *P* ≈ 31%, the spin polarization of the HR Co|h-BN injector comes out to be ≈ 65%. This spin polarization value is very high for spin injection into a non-magnetic material in general^39^, and much higher than the maximum intrinsic spin polarization of Co (≈35%^40^) and also Co|Al_2_O_3_ tunnel junctions (≈43%^41^). Thus, the large value obtained here together with the inverted sign of the signal could only be possible if significant spin filtering takes place in HR Co|h-BN|Gr contacts.

**Figure 4:**
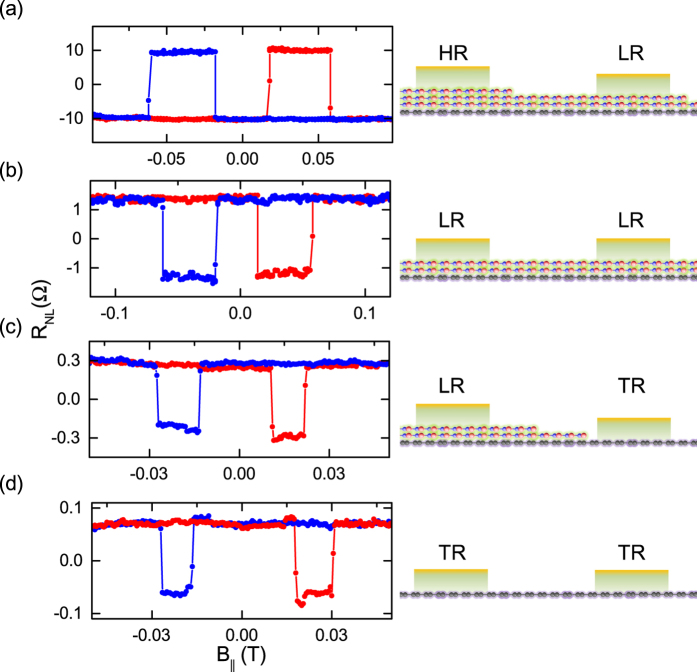
Inversion of spin valve signal with thicker h-BN tunnel barrier. Comparison of inverted and normal spin valve signals on devices with different combinations of contacts for injector and detector. All the measurements were done for an injection current of *I* = +5 μA at 300 K. Schematics of interfaces are presented for each case in the right panel. (**a)** h-BN tunnel contact with HR injector and LR detector, 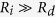 (belongs to same device as in Fig. 3a at a different injection bias). (**b)** LR h-BN tunnel contact with similar contact resistance 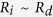. (**c)** Device with one LR h-BN tunnel contact and one transparent contact (TR). (**d)** Device with two transparent contacts (TR).

The negative spin polarization or inverted spin signal is reproducibly observed in several devices involving a high resistance h-BN tunnel contact and can originate from either of the injector or the detector contacts. In NL spin valve measurements, the usual positive spin signal sign is obtained when both the injector and detector have the same sign of spin polarization. In contrast, opposite spin polarization of the injector and detector contacts results in a negative sign of the spin valve signal. In order to trace which contact (HR or LR h-BN) is responsible for the negative sign of the spin polarization, we performed measurements on a series of devices with different combinations of tunnel resistances. In Fig. 4 we show the room temperature spin valve data for four devices with different injector-detector interface resistances: with high resistance h-BN (HR), low resistance h-BN (LR), and transparent contacts (TR). Specifically, these devices involve injector-detector combinations of HR-LR, LR-LR, LR-TR, and TR-TR.

Based on the sign of the observed spin signal, we group these devices into two categories. First kind, with inverted spin valve signal that has been observed exclusively in devices with a HR-LR contact combination (Fig. 4a), which points to opposite sign of the injector and detector polarizations. Second kind, with normal sign for spin valve signals, obtained in devices with other combinations of contact resistances as shown in Fig. 4. Although the LR-LR h-BN device shows a normal sign for a spin valve signal (Fig. 4b), with similar contact resistance and polarization, we cannot identify the sign for a LR contact here. We therefore specifically searched for LR-TR devices, where the TR contacts result from lack of local coverage of h-BN and have low resistance RA ~ 100 Ω.μm^2^. The normal sign for a spin valve signal in LR-TR combinations Fig. 4c confirms that LR h-BN contacts possess normal sign of spin polarization and cannot be responsible for the anomalous inverted spin signal observed in our HR-LR devices. We also show the normal spin valve signal obtained for the TR-TR device Fig. 4d which was prepared without h-BN. Devices using conventional oxide barriers such as TiO_2_ also show a normal spin signal without any inversion^42^. From these sets of experiments, we arrive at the conclusion that the negative spin polarization results specifically from the thicker h-BN barriers with high resistance, as shown for HR-LR devices.

**Figure 5:**
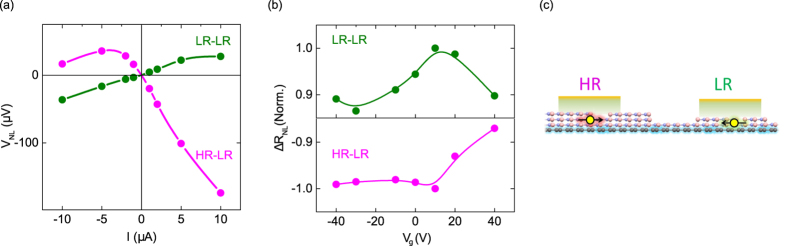
Bias and gate dependence of spin signal. (**a)** Bias dependence of the spin-valve signal at 300 K for a device with HR-LR (injector-detector) h-BN contacts and 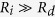 (pink) shows sign inversion. The LR-LR device with low resistance h-BN with resistances 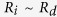 (green), shows normal sign. (**b)** A comparison of the gate voltage dependence of the nonlocal resistance for both cases. For a better comparison, the signal is normalized with the maximum spin signal in each case *ΔR_NL_*(norm) = *ΔR_NL_/ΔR_NL_*(max ≈ 20.1 Ω for HR-LR, ≈ 2.6 Ω for LR-LR). (**c)** Scheme showing opposite spin polarizations of HR and LR h-BN|Co contacts on graphene channel.

To understand the robustness of the spin signal inversion, we further studied the influence of the applied injection current bias and gate voltage on the spin signal of our devices at room temperature. Figure 5a shows a comparison of the bias dependence for two devices with HR-LR and LR-LR h-BN contact combinations. It is found that the negative sign of the spin-polarization in a HR h-BN contact persists over a wide range of the applied bias. In comparison, the LR-LR h-BN device shows a smaller and positive spin signal over the measured bias range. The spin signals display a linear dependence in the low bias range, with a non-linearity at higher bias only for the HR-LR device. Such non-linear behavior is expected because of a reduced tunnel spin polarization of Co|h-BN contacts at higher bias as discussed before. Notably, we did not observe any change in the sign of the spin polarization with applied bias in either of the devices in the measured bias range. These results indicate that the sign of the spin polarization of the Co|h-BN contacts is robust, and rules out the possibility of using the bias to tune the accessible density of states from majority to minority or vice-versa^43^.

Next, the effect of a gate voltage (*V_g_*), which controls the carrier density in graphene, on the sign of the spin signal is examined. In Fig. 5b, we show the normalized spin signal *ΔR_NL_* for both HR-LR and LR-LR devices in the range *V_g_* = ±40 V. In both cases we observe a decrease in the magnitude of *ΔR_NL_* by 10–15% for higher *V_g_* (higher carrier concentration). This behavior is in agreement with theory^44^ and experiments^45^, where tunnel resistance *R_T_* is much higher than the graphene channel resistance *R_Ch_*. In such regime, a decreasing trend in the magnitude of *ΔR_NL_* at higher gate voltage *V_g_* is usually observed in lateral spin valve devices. Throughout the *V_g_* sweep range, we observe a negative *ΔR_NL_* for the HR-LR device, which shows that the observed negative spin polarization in a HR Co|h-BN|Gr tunnel contact is stable against variation of carrier density in graphene.

## Discussion

Spin resolved conductance across the junctions of 2D crystals with ferromagnets has been the subject of recent theoretical and experimental interest due to their atomically thin nature. Karpan *et al.* first proposed strong spin resolved conductance leading to a perfect spin filtering in FM|graphene (graphite) interfaces^6^, signatures of which were found experimentally^46^,^47^,^48^. Next, several theoretical calculations have been also performed to understand the spin resolved conductance across FM|h-BN junctions. Karpan *et al.* predicted^7^ that for 1–4 atomic layers of h-BN the minority spin transport channel (weakly) dominates transport though the FM|h-BN interfaces giving rise to a thickness dependent negative spin polarization. Similar studies for a monolayer and bilayer h-BN junctions have revealed the spin-resolved quantum conductance of the minority spins to dominate over the majority spins leading to minority spin polarization irrespective of the Co crystal structure, fcc or hcp^8^,^24^. In addition, calculations on FM|h-BN|Gr junctions also showed minority spin dominated conduction showing enhanced polarization with thicker h-BN in such junctions^23^. The density of states (DoS) at the Fermi level of strong ferromagnetic metals such as Co or Ni are dominated by the minority spin channel that theoretically leads to a negative DoS polarization. Although, h-BN by itself has no spin dependent conductance, the transmission through a FM|h-BN|Gr junction is spin dependent and depends upon the thickness of h-BN in the junctions that can increase the spin injection efficiency with overall negative polarization of such contacts. Our results on HR h-BN tunnel contacts with enhanced values of negative spin polarization are consistent with theoretical results. However, the positive spin polarization in LR contacts is similar to traditional oxide barrier interfaces. Experimentally ferromagnetic tunnel contacts with tunnel barriers of traditional oxide such as Al_2_O_3_, MgO have been observed to bear a positive spin polarization^49^, while contacts with Al_2_O_3_-Ta_2_O_3_, SrRuO_3_ and Fe_3_O_4_ barriers have also shown negative spin polarization^43^,^50^,^51^,^52^,^53^. Such variance has been attributed to the unique spin dependent properties of complex electronic structure of the ferromagnet-barrier hybrid interface. In our experiments, the difference of sign of spin polarization of HR and LR h-BN contacts could possibly stem from a significant different interface conditions. A thicker HR barrier that can alleviate the problems of doping and diffusion ferromagnetic atoms into the barrier and graphene. Unlike this, a thinner LR h-BN barrier is much more susceptible to lose structural integrity and more prone to doping leading doping induced interface states and defects^53^,^54^ and a much more complex interface structure. In such interfaces with defects, it is known that resonant tunneling via defect states can invert spin polarization^54^. Even though processes like multistep tunneling are known to decrease the spin polarization effectively leading to lower spin signal, the ultralow thicknesses of HR and LR barriers make it extremely unlikely and could be ruled out^55^. We acknowledge that a detailed and quantitative understanding of the actual mechanism dominating at the interface needs to be understood by more theoretical and experimental studies. Nevertheless, our results and controlled experiments with different combinations of barrier along with previous theoretical predictions in the literature indicate spin filtering across HR Co|h-BN|Gr junctions leading to large negative spin polarization.

## Summary

In conclusion, we have observed an inversion of spin signal in non-local graphene spin valves with ferromagnetic tunnel contacts with h-BN barrier. Through controlled experiments on several devices having different injector-detector contact resistances, we confirm that the high resistance Co|h-BN junctions with negative spin polarization lead to the observed inversion of spin signal. The effect is found to be robust as established through systematic bias and gate voltage dependent measurements. The enhanced polarization with thicker h-BN junctions indicates considerable spin filtering of spatial nature. These findings bring out the significance of CVD h-BN as a novel spin tunnel barrier, well beyond its use as a 2D insulating barrier that can provide interface resistance for addressing the conductivity mismatch problem. It also demonstrates the possibility of achieving different polarization with different thicknesses of h-BN offering different spin dependent interface conditions and spin filtering. The possibility of high spin polarization in thicker multilayer h-BN together with better quality graphene would be a key ingredient for efficiency in future graphene spintronic devices. In addition, the novel spin filtering effects of ferromagnetic tunnel contacts with thicker h-BN barriers can be explored for engineering spintronic devices with other 2D crystals^56^,^57^,^58^ and their van der Waals heterostructures.

## Methods

The Graphene devices with Co|h-BN tunnel contacts were prepared on a highly doped Si substrate with 285 nm SiO_2_. Graphene flakes are exfoliated on a cleaned SiO_2_/Si substrate with predefined Ti/Au markers by repeated peeling of highly oriented pyrolytic graphite (HOPG) using the conventional scotch tape technique. The graphene flakes were selected using a combination of optical and atomic-force microscopy. The CVD h-BN layer used as tunnel barrier in our experiment, was grown by chemical vapor deposition (CVD) on a copper foil and has high crystalline quality with ~90% coverage (procured from Graphene supermarket). The CVD h-BN is placed on graphene by wet chemical etching and transfer processes, respectively. The h-BN surface is first covered with PMMA, and then isolated from the Cu substrate by etching in a FeCl_3_ solution. The h-BN/PMMA layer is washed with 10% HCl and deionized water, and subsequently transferred onto the chip containing graphene flakes. The chip is then baked at 150 °C and washed with acetone to remove the PMMA. The resulting chip containing a h-BN|graphene heterostructure is annealed at 400° C in Ar/H_2_ atmosphere. The annealing improves the adhesion between the h-BN and graphene and ensures the removal of possible residues of resist remaining from the transfer process of h-BN. The h-BN layer is patterned in regions covering graphene flakes by electron beam lithography and subsequent Ar-etching. In the 2^nd^ step of e-beam lithography, electrodes were patterned and cobalt of 65 nm deposited by electron beam evaporation at a rate of 2 Å/sec at a pressure of <5 × 10^−7^ mbar with sample being placed in normal position without any specific tilt of the sample stage. The ferromagnetic Co deposition was followed by deposition of 20 nm thick Au capping layer without breaking the vacuum to protect them from possible oxidation. The electrical and spin transport measurements were performed in a liquid helium cryostat with a superconducting magnet.

## Additional Information

**How to cite this article**: Kamalakar, M. V. *et al.* Inversion of Spin Signal and Spin Filtering in Ferromagnet|Hexagonal Boron Nitride-Graphene van der Waals Heterostructures. *Sci. Rep.* **6**, 21168; doi: 10.1038/srep21168 (2016).

## Supplementary Material

Supplementary Information

## References

[b1] Novoselov K. S. *et al.* Two-dimensional atomic crystals. Proc. Natl. Acad. Sci. USA 102, 10451–10453 (2005). 16027370 10.1073/pnas.0502848102 PMC1180777

[b2] Britnell L. *et al.* Electron tunneling through ultrathin boron nitride crystalline barriers. Nano Lett. 12, 1707–1710 (2012). 22380756 10.1021/nl3002205

[b3] Britnell L. *et al.* Field-effect tunneling transistor based on vertical graphene heterostructures. Science 335, 947–50 (2012). 22300848 10.1126/science.1218461

[b4] Tombros N., Jozsa C., Popinciuc M., Jonkman H. T. & van Wees B. J. Electronic spin transport and spin precession in single graphene layers at room temperature. Nature 448, 571–574 (2007). 17632544 10.1038/nature06037

[b5] Yamaguchi T. *et al.* Electrical Spin Injection into Graphene through Monolayer Hexagonal Boron Nitride. Appl. Phys. Express 6, 073001 (2013).

[b6] Karpan V. M. *et al.* Graphite and Graphene as Perfect Spin Filters. Phys. Rev. Lett. 99, 176602 (2007). 17995355 10.1103/PhysRevLett.99.176602

[b7] Karpan V. M., Khomyakov P. A., Giovannetti G., Starikov A. A. & Kelly P. J. Ni(111)|graphene|h-BN junctions as ideal spin injectors. Phys. Rev. B 84, 153406 (2011).

[b8] Yazyev O. & Pasquarello A. Magnetoresistive junctions based on epitaxial graphene and hexagonal boron nitride. Phys. Rev. B 80, 035408 (2009).

[b9] Kamalakar M. V., Dankert A., Bergsten J., Ive T. & Dash S. P. Enhanced Tunnel Spin Injection into Graphene using Chemical Vapor Deposited Hexagonal Boron Nitride. Sci. Rep. 4, 6146 (2014). 25156685 10.1038/srep06146 PMC4143790

[b10] Karpan V. M. *et al.* Theoretical prediction of perfect spin filtering at interfaces between close-packed surfaces of Ni or Co and graphite or graphene. Phys. Rev. B 78, 195419 (2008).

[b11] Kamalakar M. V., Groenveld C., Dankert A. & Dash S. P. Long Distance Spin Communication in Chemical Vapour deposited Graphene. Nat. Commun. 6, 6766 (2015). 25857650 10.1038/ncomms7766 PMC4433146

[b12] Dean C. R. *et al.* Boron nitride substrates for high-quality graphene electronics. Nat. Nanotechnol. 5, 722–726 (2010). 20729834 10.1038/nnano.2010.172

[b13] Fu W., Makk P., Maurand R., Bräuninger M. & Schönenberger C. Large-scale BN tunnel barriers for graphene spintronics. J. Appl. Phys. 116, 074306 (2014).

[b14] Geim A. K. & Grigorieva I. V. Van der Waals heterostructures. Nature 499, 419–25 (2013). 23887427 10.1038/nature12385

[b15] Zomer P. J., Guimarães M. H. D., Tombros N. & van Wees B. J. Long-distance spin transport in high-mobility graphene on hexagonal boron nitride. Phys. Rev. B 86, 161416 (2012).

[b16] Drögeler M. *et al.* Nanosecond spin lifetimes in single- and few-layer graphene-hBN heterostructures at room temperature. Nano Lett. 14, 6050–5 (2014). 25291305 10.1021/nl501278c

[b17] Giuimaraes M. H. D. *et al.* Controlling Spin Relaxation in Hexagonal BN-Encapsulated Graphene with a Transverse Electric Field. Phys. Rev. Lett. 113, 086602 (2014). 25192116 10.1103/PhysRevLett.113.086602

[b18] Ingla-Aynés J., Guimarães M. H. D., Meijerink R. J., Zomer P. J. & van Wees B. J. 24 − μ m spin relaxation length in boron nitride encapsulated bilayer graphene. Phys. Rev. B 92, 201410 (2015).

[b19] Fu W., Makk P., Bräuninger M., Maurand R. & Schönenberger C. Large-scale fabrication of BN tunnel barriers for graphene spintronics. J. Appl. Phys. 116, 074306 (2014).

[b20] Kamalakar M. V., Dankert A., Bergsten J., Ive T. & Dash S. P. Spintronics with graphene-hexagonal boron nitride van der Waals heterostructures. Appl. Phys. Lett. 105, 212405 (2014).

[b21] Dankert A., Kamalakar M. V., Wajid A., Patel R. S. & Dash S. P. Tunnel magnetoresistance with atomically thin two dimensional hexagonal boron nitride barriers. Nano Res. 8, 1357–1364 (2014).

[b22] Fert A. & Jaffrès H. Conditions for efficient spin injection from a ferromagnetic metal into a semiconductor. Phys. Rev. B 64, 184420 (2001).

[b23] Wu Q. *et al.* Efficient Spin Injection into Graphene through a Tunnel Barrier: Overcoming the Spin-Conductance Mismatch. Phys. Rev. Appl. 2, 044008 (2014).

[b24] Hu M. L., Yu Z., Zhang K. W., Sun L. Z. & Zhong J. X. Tunneling Magnetoresistance of Bilayer Hexagonal Boron Nitride and Its Linear Response to External Uniaxial Strain. J. Phys. Chem. C 115, 8260–8264 (2011).

[b25] Jönsson-Åkerman B. J. *et al.* Reliability of normal-state current–voltage characteristics as an indicator of tunnel-junction barrier quality. Appl. Phys. Lett. 77, 1870 (2000).

[b26] Lee, J.-H. *et al.* Wafer-scale growth of single-crystal monolayer graphene on reusable hydrogen-terminated germanium. Science 344, 286–9 (2014). 24700471 10.1126/science.1252268

[b27] Volmer F. *et al.* Role of MgO barriers for spin and charge transport in Co/MgO/graphene nonlocal spin-valve devices. Phys. Rev. B 88, 161405 (2013).

[b28] Dlubak B. *et al.* Highly efficient spin transport in epitaxial graphene on SiC. Nat. Phys. 8, 557–561 (2012).

[b29] Fert A., George J. M., Jaffrès H. & Mattana R. Semiconductors between spin-polarized sources and drains. IEEE Trans. Electron Devices 54, 921–932 (2007).

[b30] Maassen T., Vera-Marun I. J., Guimarães M. H. D. & van Wees B. J. Contact-induced spin relaxation in Hanle spin precession measurements. Phys. Rev. B 86, 235408 (2012).

[b31] Idzuchi H., Fukuma Y., Takahashi S., Maekawa S. & Otani Y. Effect of anisotropic spin absorption on the Hanle effect in lateral spin valves. Phys. Rev. B 89, 081308 (2014).

[b32] Han W. *et al.* Spin transport and relaxation in graphene. J. Magn. Magn. Mater. 324, 369–381 (2012).

[b33] Friedman A. L., van ’t Erve O. M. J., Li C. H., Robinson J. T. & Jonker B. T. Homoepitaxial tunnel barriers with functionalized graphene-on-graphene for charge and spin transport. Nat. Commun. 5, 3161 (2014). 24445349 10.1038/ncomms4161

[b34] Moodera J., Nowak J. & van de Veerdonk R. Interface Magnetism and Spin Wave Scattering in Ferromagnet-Insulator-Ferromagnet Tunnel Junctions. Phys. Rev. Lett. 80, 2941–2944 (1998).

[b35] Park B., Banerjee T., Lodder J. & Jansen R. Tunnel Spin Polarization Versus Energy for Clean and Doped Al2O3 Barriers. Phys. Rev. Lett. 99, 217206 (2007). 18233249 10.1103/PhysRevLett.99.217206

[b36] Valenzuela S., Monsma D., Marcus C., Narayanamurti V. & Tinkham M. Spin Polarized Tunneling at Finite Bias. Phys. Rev. Lett. 94, 196601 (2005). 16090193 10.1103/PhysRevLett.94.196601

[b37] Yuasa S., Nagahama T., Fukushima A., Suzuki Y. & Ando K. Giant room-temperature magnetoresistance in single-crystal Fe/MgO/Fe magnetic tunnel junctions. Nat. Mater. 3, 868–871 (2004). 15516927 10.1038/nmat1257

[b38] LeClair P. *et al.* Band Structure and Density of States Effects in Co-Based Magnetic Tunnel Junctions. Phys. Rev. Lett. 88, 107201 (2002). 11909383 10.1103/PhysRevLett.88.107201

[b39] Idzuchi H., Fukuma Y. & Otani Y. Spin transport in non-magnetic nano-structures induced by non-local spin injection. Phys. E Low-dimensional Syst. Nanostructures 68, 239–263 (2015).

[b40] Tedrow P. & Meservey R. Spin Polarization of Electrons Tunneling from Films of Fe, Co, Ni, and Gd. Phys. Rev. B 7, 318–326 (1973).

[b41] Monsma D. J. & Parkin S. S. P. Spin polarization of tunneling current from ferromagnet/Al_2_O_3_ interfaces using copper-doped aluminum superconducting films. Appl. Phys. Lett. 77, 720 (2000).

[b42] Dankert A., Kamalakar M. V., Bergsten J. & Dash S. P. Spin transport and precession in graphene measured by nonlocal and three-terminal methods. Appl. Phys. Lett. 104, 192403 (2014).

[b43] Sharma M., Wang S. & Nickel J. Inversion of Spin Polarization and Tunneling Magnetoresistance in Spin-Dependent Tunneling Junctions. Phys. Rev. Lett. 82, 616–619 (1999).

[b44] Takahashi S. & Maekawa S. Spin injection and detection in magnetic nanostructures. Phys. Rev. B 67, 052409 (2003).

[b45] Han W. *et al.* Tunneling Spin Injection into Single Layer Graphene. Phys. Rev. Lett. 105, 167202 (2010). 21231003 10.1103/PhysRevLett.105.167202

[b46] Dlubak B. *et al.* Graphene-passivated nickel as an oxidation-resistant electrode for spintronics. ACS Nano 6, 10930–4 (2012). 23145543 10.1021/nn304424x

[b47] Singh A. K. & Eom J. Negative magnetoresistance in a vertical single-layer graphene spin valve at room temperature. ACS Appl. Mater. Interfaces 6, 2493–6 (2014). 24495123 10.1021/am4049145

[b48] Godel F. *et al.* Voltage-controlled inversion of tunnel magnetoresistance in epitaxial nickel/graphene/MgO/cobalt junctions. Appl. Phys. Lett. 105, 152407 (2014).

[b49] Swagten H. J. M. Spin-Dependent Tunneling in Magnetic Junctions. Handb. Magn. Mater. 17, 1–121 (2007).

[b50] Teresa J. M. De *et al.* Role of Metal-Oxide Interface in Determining the Spin Polarization of Magnetic Tunnel Junctions. Science (80-.). 286, 507–509 (1999). 10.1126/science.286.5439.507 10521341

[b51] Worledge D. C. & Geballe T. H. Negative Spin-Polarization of SrRuO3. Phys. Rev. Lett. 85, 5182–5185 (2000). 11102216 10.1103/PhysRevLett.85.5182

[b52] Hu G. & Suzuki Y. Negative Spin Polarization of Fe3O4 in Magnetite/Manganite-Based Junctions. Phys. Rev. Lett. 89, 276601 (2002). 12513228 10.1103/PhysRevLett.89.276601

[b53] Tsymbal E. *et al.* Interface effects in spin-dependent tunneling. Prog. Mater. Sci. 52, 401–420 (2007).

[b54] Tsymbal E., Sokolov a., Sabirianov I. & Doudin B. Resonant Inversion of Tunneling Magnetoresistance. Phys. Rev. Lett. 90, 186602 (2003). 12786034 10.1103/PhysRevLett.90.186602

[b55] Tran T. L. A., Le T. Q., Sanderink J. G. M., Van Der Wiel W. G. & De Jong M. P. The multistep tunneling analogue of conductivity mismatch in organic spin valves. Adv. Funct. Mater. 22, 1180–1189 (2012).

[b56] Dankert A., Langouche L., Kamalakar M. V. & Dash S. P. High-performance molybdenum disulfide field-effect transistors with spin tunnel contacts. ACS Nano 8, 476–82 (2014). 24377305 10.1021/nn404961e

[b57] Kamalakar M. V., Madhushankar B. N., Dankert A. & Dash S. P. Low Schottky Barrier Black Phosphorus Field-Effect Devices with Ferromagnetic Tunnel Contacts. Small 11, 2209–2216 (2015). 25586013 10.1002/smll.201402900

[b58] Dankert A., Geurs J., Kamalakar M. V., Charpentier S. & Dash S. P. Room Temperature Electrical Detection of Spin Polarized Currents in Topological Insulators. Nano Lett. (2015). 10.1021/acs.nanolett.5b03080 26560203

