# Supplementary material for: Inversion of Spin Signal and Spin Filtering in Ferromagnet|Hexagonal Boron Nitride-Graphene van der Waals Heterostructures
Source: Sci Rep. 2016 Feb 17;6:21168. doi: 10.1038/srep21168 (PMC4756790; doi:10.1038/srep21168)
Supplement: Supplementary Information [file srep21168-s1.pdf]

Supplementary Information

Inversion of Spin Signal and Spin Filtering in Ferromagnet|Hexagonal Boron Nitride-Graphene van der Waals Heterostructures

M. Venkata Kamalakar[1,2], André Dankert[1], Paul J. Kelly[3] and Saroj P. Dash[1†]*

[1] Department of Microtechnology and Nanoscience, Chalmers University of Technology, SE-41296, Göteborg, Sweden.

[2]Department of Physics and Astronomy, Uppsala University, Box 516, 75120, Uppsala, Sweden

[3] Faculty of Science and Technology and MESA[+] Institute for Nanotechnology, University of Twente, P.O. Box 217, 7500 AE Enschede, The Netherlands.

**venkata.mutta@physics.uu.se; †saroj.dash@chalmers.se*

1. CVD h-BN coverage

After the transfer process of CVD h-BN to the SiO_2/Si substrate (see experimental section in the main manuscript), we observed very good coverage of the layer (Fig. S1), with h-BN absent only at few places.

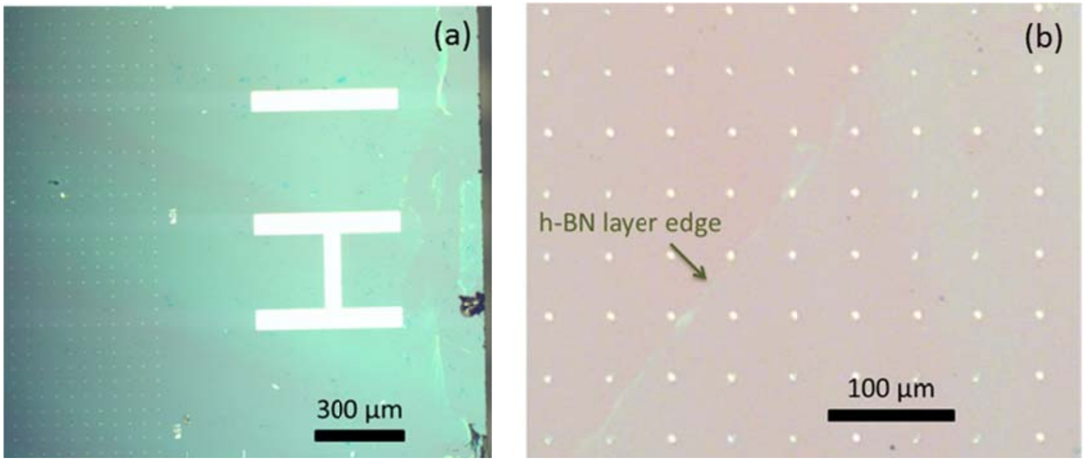

Figure S1: *(a) Optical microscope image showing chip scale ripple free transfer of CVD h-BN to SiO_2/Si substrate. (b) Magnified view showing the uniform coverage of CVD h-BN.*

From atomic force microscopy performed on processed devices (shown in Fig. S2), we found the thickness of the layer varied mostly between 5-10 Å on SiO_2 substrate, which corresponds to between 1-3 atomic layers of h-BN[1]. This is what gives rise to the variation of tunnel contact resistance in our devices. The details of such variation with resistance scaling have been elucidated by previous reports[2,3]. It should be noted that the thickness of h-BN measured with AFM is the effective thickness obtained on SiO_2 substrate, and not the

exact thickness of h-BN layers. Thus, both AFM and contact resistance measurements together provide a good estimate of the CVD h-BN layer thickness.

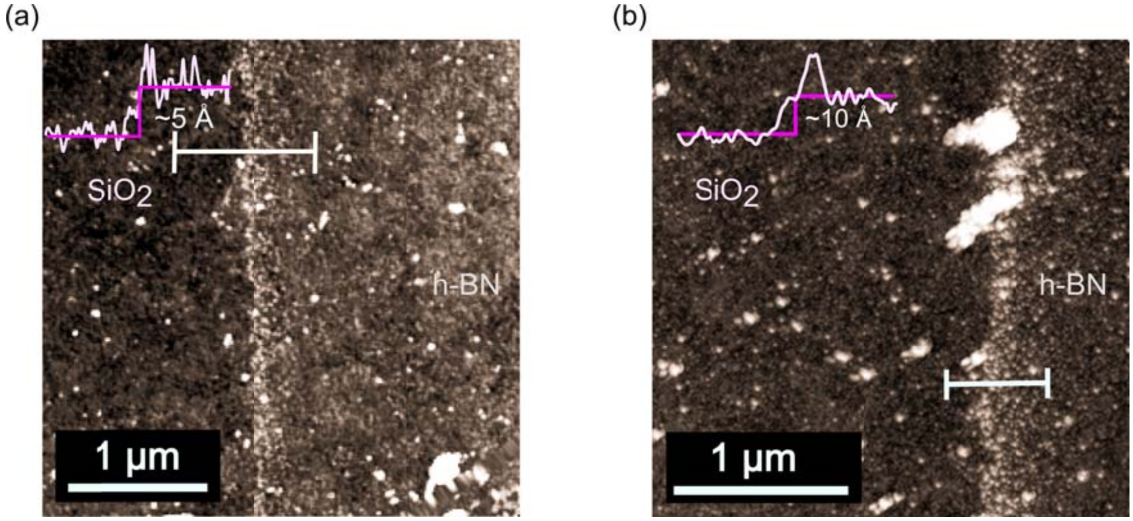

Figure S2: *(a), (b) Atomic force microscopy images of CVD h-BN on SiO_2 substrate performed on fabricated devices, showing effective thickness of 5 – 10* Å.

In the above process of CVD h-BN transfer and fabrication of ferromagnetic tunnel contacts, we took extreme care to avoid any residues on the surface and interfaces. We identified and optimized the following two key processes which results in negligible influence of resist residues. First, we developed the method of room temperature curing of the support PMMA resist layer for one day during the transfer process. This greatly improved the transfer process and minimized polymer residues. After transferring the h-BN layer, we annealed the chip in an Ar/H_2 atmosphere at 400°C to further remove any resist residues. The annealing improves the adhesion between the h-BN and graphene and ensures the removal of possible residues of resist remaining from the transfer process of h-BN. Second, while pattering the h-BN layer by etching with an Ar plasma, PMMA resist edges patterned by e-beam lithography can harden. Again, we identified that excessive Ar plasma etching can harden the resist leading to possible residues at the edges of patterns. We optimized both processes to achieve clean interfaces. The electrodes of ferromagnetic Co with a capping Au layer are prepared on the h-BN/graphene heterostructures by electron beam lithography and lift off techniques. Furthermore, electrical measurement on such fabricated devices showed reproducible tunneling behavior and regular enhancement of spin signal with higher tunnel contact resistance (as shown in Fig. 4 and presented in our previous report[2,3]). On the other hand, contacts prepared using a conventional transfer process that involves resist residues showed unstable contact resistances and poor or no spin signal, often disproportionate to their expected levels.

2. Spin transport with normal sign of spin signal

We obtained normal sign for spin valve and Hanle spin signal (Fig. S3) in all devices having low resistance h-BN contacts for both injector and detector contacts.

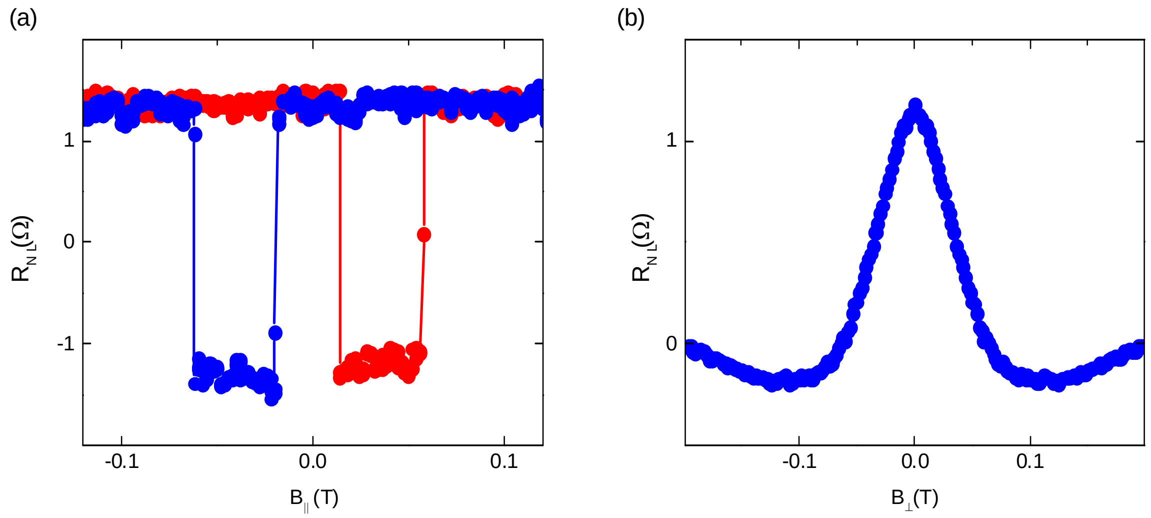

Figure S3: *Spin transport and precession measurements at room temperature with low resistance (LR) h-BN/Co contacts for both injector and detector having normal sign of spin signal in nonlocal geometry* ***(a)*** *Spin valve and* ***(b)*** *Hanle curve.*

3. Spin lifetime and diffusion constant

The spin lifetime and diffusion constant in this study shown in Fig. S4 were evaluated from different devices having different contact resistances, prepared on the same substrate and on graphene flakes with similar electrical properties.

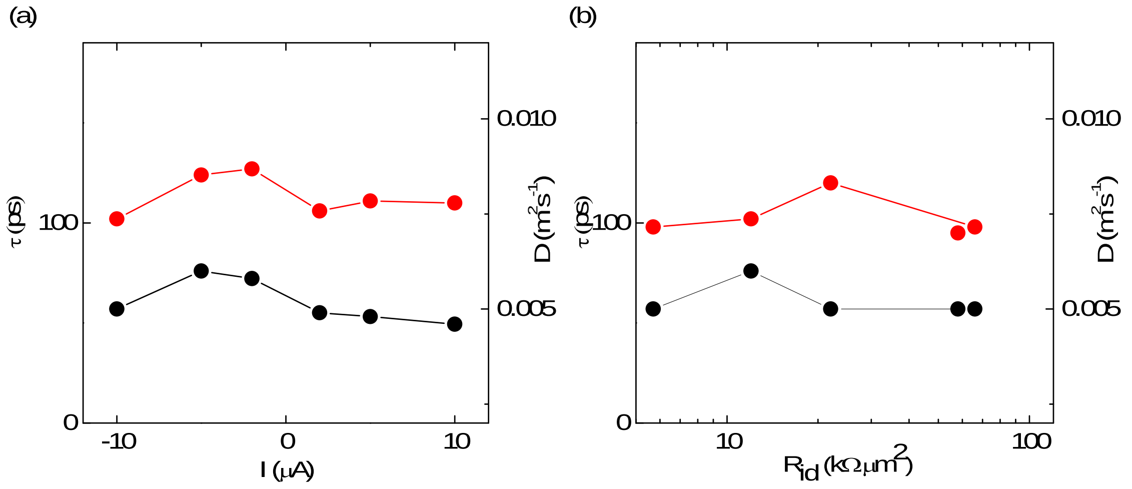

Figure S4: ***(a)*** *Injection bias current dependence of spin lifetime* τ_s *and diffusion constant* D_s. ***(b)*** *spin lifetime* τ_s *and diffusion constant* D_s *as a function of contact resistance* $R_{id} = \sqrt{R_i R_d}$ *as obtained on different devices:* $R_i \rightarrow$ *injector,* $R_d \rightarrow$ *detector.*

4. Electrical characterization of graphene channel

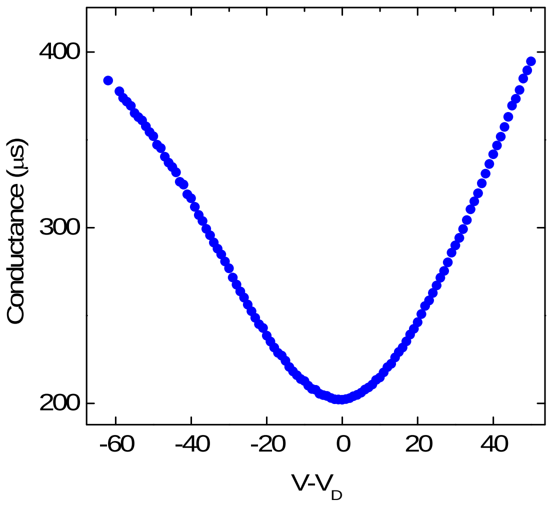

Figure S5: *Dirac curve of the graphene channel of the device presented in Fig. 3a, b of main text.*

We characterized the graphene channel by varying the back gate voltage for the device presented in the main text (Fig. 3a, b). Figure S5 shows the Dirac curve measured in a four probe geometry at room temperature. We estimate the channel mobility to be μ ~ 1000 $cm^2 V^{-1} s^{-1}$. The relatively low mobility that we obtained in this device possibly leads to the low spin relaxation time of 100 ps.

5. Magnetoresistance calculation

In order to show a comparison of h-BN tunnel contact resistance and graphene spin resistance in our devices, in Fig. S6 we show the magnetoresistance calculated using Fert- Jaffrès model for graphene case[4]. In our devices the tunnel resistance R_T of h-BN contacts lie in the region with green shade much higher than the graphene channel spin resistance R_{ch}^s, which is required for efficient spin injection.

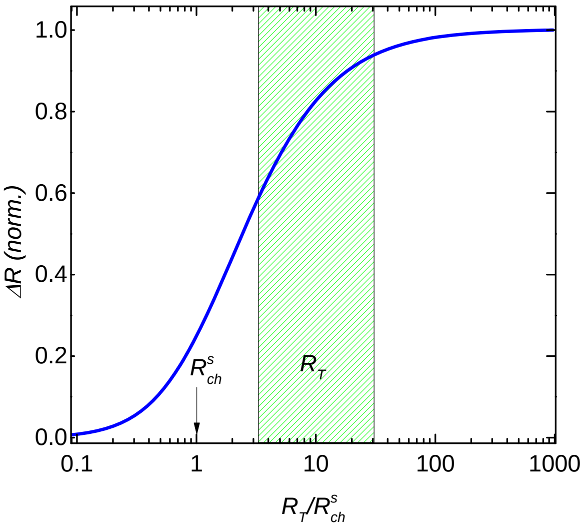

Figure S6: Calculated magnetoresistance (normalized) as a function of tunnel barrier resistance R_T/R_{ch}^s.

References

(1) Britnell, L.; Gorbachev, R. V; Jalil, R.; Belle, B. D.; Schedin, F.; Katsnelson, M. I.; Eaves, L.; Morozov, S. V; Mayorov, A. S.; Peres, N. M. R.; *et al.* Electron Tunneling through Ultrathin Boron Nitride Crystalline Barriers. *Nano Lett.* **2012**, *12*, 1707–1710.

(2) Kamalakar, M. V.; Dankert, A.; Bergsten, J.; Ive, T.; Dash, S. P. Enhanced Tunnel Spin Injection into Graphene Using Chemical Vapor Deposited Hexagonal Boron Nitride. *Sci. Rep.* **2014**, *4*, 6146.

(3) Fu, W.; Makk, P.; Maurand, R.; Bräuninger, M.; Schönenberger, C. Large-Scale BN Tunnel Barriers for Graphene Spintronics. *J. Appl. Phys.* **2014**, *116*, 074306.

(4) Dlubak, B.; Martin, M.-B.; Deranlot, C.; Servet, B.; Xavier, S.; Mattana, R.; Sprinkle, M.; Berger, C.; De Heer, W. a.; Petroff, F.; *et al.* Highly Efficient Spin Transport in Epitaxial Graphene on SiC. *Nat. Phys.* **2012**, *8*, 557–561.
